# Physiological Responses and Physical Performance during Football in the Heat

**DOI:** 10.1371/journal.pone.0039202

**Published:** 2012-06-19

**Authors:** Magni Mohr, Lars Nybo, Justin Grantham, Sebastien Racinais

**Affiliations:** 1 Sport and Health Sciences, College of Life and Environmental Sciences, St. Lukes Campus, University of Exeter, Exeter, United Kingdom; 2 Department of Exercise and Sport Sciences, Section of Human Physiology University of Copenhagen, Copenhagen, Denmark; 3 Aspetar, Qatar Orthopaedic and Sports Medicine Hospital, Research and Education Centre, Doha, Qatar; Instituto de Investigación Hospital 12 de Octubre, Spain

## Abstract

**Purpose:**

To examine the impact of hot ambient conditions on physical performance and physiological responses during football match-play.

**Methods:**

Two experimental games were completed in temperate (∼21°C; CON) and hot ambient conditions (∼43°C; HOT). Physical performance was assessed by match analysis in 17 male elite players during the games and a repeated sprint test was conducted after the two game trials. Core and muscle temperature were measured and blood samples were obtained, before and after the games.

**Results:**

Muscle and core temperatures were ∼1°C higher (P<0.05) in HOT (40.3±0.1 and 39.5±0.1°C, respectively) compared to CON (39.2±0.1 and 38.3±0.1°C). Average heart rate, plasma lactate concentration, body weight loss as well as post-game sprint performance were similar between the two conditions. Total game distance declined (P<0.05) by 7% and high intensity running (>14 km⋅h^−1^) by 26% in HOT compared to CON), but peak sprint speed was 4% higher (P<0.05) in HOT than in CON, while there were no differences in the quantity or length of sprints (>24 km⋅h^−1^) between CON and HOT. In HOT, success rates for passes and crosses were 8 and 9% higher (P<0.05), respectively, compared to CON. Delta increase in core temperature and absolute core temperature in HOT were correlated to total game distance in the heat (r = 0.85 and r = 0.53, respectively; P<0.05), whereas, total and high intensity distance deficit between CON and HOT were not correlated to absolute or delta changes in muscle or core temperature.

**Conclusion:**

Total game distance and especially high intensity running were lower during a football game in the heat, but these changes were not directly related to the absolute or relative changes in core or muscle temperature. However, peak sprinting speed and execution of successful passes and crosses were improved in the HOT condition.

## Introduction

Playing football in the heat has been shown to result in high body temperatures [1]–[3], which may have a negative impact on performance and accelerate the development of fatigue during a game. While the total distance covered in a football match may be lower in relation to temperate conditions even in moderate heat [1], the total game-distance alone has been shown to be a poor football match-performance indicator [4], [5]. With regards to sprint performance, maximal sprint performance may be improved when the muscle temperature is elevated prior to competition [6], whereas the ability to perform repeated high intensity exercise has been reported to deteriorate with hyperthermia even though higher muscle temperatures are reached [7], [8]. A possible explanation may be that hyperthermia limits whole-body exercise even before peripheral muscle factors became limiting [9], [10], [11]. Thus, hyperthermia appears to affect various physiological systems and types of physical performance components in a complex manner, making its net-effect on a multi-faceted activity like football difficult to resolve.

Only two studies have compared the effect of elevated ambient temperature on match performance in football. Ekblom [1] compared a game at moderate heat (30°C) to a game at a normal temperature (20°C), while Ozgunen et al. [3] compared two games in the heat with only two degrees difference. Neither of these studies could elucidate the physiological and performance responses of athletes normally competing in temperate conditions when exposed to severe heat stress (>40°C) which often occurs during international tournaments and will be expected at the upcoming football World Cup in Qatar in 2022. Mohr et al. [2] have reported that in the last 15 minutes of a football match played in moderately high environmental temperatures (∼30°C), high intensity running decreased markedly and the muscle temperature in some players was in excess of 41°C at the end of the match. Therefore, it appears that the ability to perform high intensity running towards the end of a match may be more affected in the heat compared to observations from temperate conditions. However, the mechanisms for this response have not been elucidated or directly compared to a matched control situation.

In an integrated physiological system, fatigue/altering the pacing strategy in hyperthermic settings appears to be mediated by a complex interplay between peripheral, central and perceptual mechanisms [9]–[11], as well as anticipatory responses [12]. However, hyperthermia-induced performance decrement has been suggested to be directly affected by an elevated cerebral temperature that may provoke central fatigue [13], [14]. Fatigue during exercise has been associated with numerous physiological impairments and is highly dependent on the type of exercise performed [10]. In football, where prolonged intermittent exercise is combined with multiple sprints, high intensity actions and challenging co-ordination tasks, fatigue is a complicated phenomenon involving interplay between several physiological mechanisms [15], [16]. Therefore we hypothesized that the different components of football performance would respond differently to heat stress. Thus, the present study investigated the effect of environmental heat stress on body temperatures and activity and performance patterns during a football game. This was achieved by having elite Scandinavian football players, unfamiliar with exercising in severe heat stress, play a control game (CON) in a temperate environment (∼21°C) and a game in hot ambient conditions (HOT; ∼43°C).

## Materials and Methods

### Ethical Statement

All subjects were informed of potential risks and discomforts associated with the experiment before giving their written consent to participate. The study conformed to the code of Ethics of World Medical Association (Declaration of Helsinki) and was approved by the Research Committee from Aspetar, Qatar Orthopaedic and Sports Medicine Hospital in Doha, Qatar.

### Subjects

Seventeen of the 20 outfield players from two teams played the full 90 minutes in the two game trials and were included in this study. They were elite male football players (age: 26.6±1.2 yrs; height: 1.84±0.01 cm; body mass: 80.1±1.6 kg) from two Scandinavian countries (Faroe Islands and Denmark). Collectively, the players encompassed all outfield playing positions and a Yo-Yo intermittent recovery level 1 (Yo-Yo IR1) performance of 2236±57 m, which was completed as part of the experimental testing, and indicates that the participants were well-trained [4], [17].

### Experimental Design

Two experimental football game trials separated by six days were completed towards the end of the competitive season. The first experimental game (control; CON) took place at normal environmental temperature (21°C) in an indoor hall (Aspire Dome, Doha, Qatar) while the second game was played outside in a hot environment (43°C; HOT). Relative humidity during the two games was 55 and 12%, respectively. The training and preparation routines were identical prior to the two game trials. Both games were played on artificial grass on pitches with similar dimensions at the same time of the day. The pre-game and half-time procedures, as well as the coaching during the game, were similar to competitive game scenarios. The same match officials controlled both games. Twenty outfield players were part of the study initially, however, due to injuries, only 17 players completed both game trials. Substitute players that were not participating as subjects replaced the injured players. During the entirety of both games the players’ activity profile was determined by a multiple-camera tracking system. Baseline repeated sprint tests were completed prior to the experimental games in resting condition as well as after both games (all repeated sprint tests were performed on the same indoor track, in a thermo-neutral environment). Finally, physiological measurements were performed prior to and after the CON and HOT games.

### Match Analysis Procedures

The players’ work profile was determined by the Amisco® multiple-camera semi-automatic passive tracking system [18], [19]. Data for total distance covered, high-intensity running (>14 km⋅h^−1^) and sprinting (>24 km⋅h^−1^) was computed in 5 and 15-minute intervals, as well as for each half and the entire game. Two permanent game fatigue indexes (P-FI; [2]) were calculated as the percentage difference in high intensity running between the first (0–15 min) and the last (75–90 min) 15-minute interval of the game (P-FI_1_; see also [2]) and the percentage difference between the average high intensity running distance in the first five 15-minute periods (from 0–75 min) and the final 15-minutes (75–90 min) of the game (P-FI_2_). In addition, a temporary game-fatigue index (T-FI) was calculated based on the percentage difference between amounts of high-intensity running during the most intense 5-minute period and the 5-minute period following the most intense period (see also [4]). Finally, data on sprint characteristics during the game such as average and peak sprinting speed and sprint length were recorded.

### Amisco® Multiple-camera System

The Amisco® system is a multiple-camera match analysis system (Amisco Pro®, version 1.0.2, Nice, France). The movements of all outfield players were recorded during the game by three stable synchronized cameras positioned at a height of approximately 10 m above the pitch and sampled data at a frequency of 25 Hz. Signals and angles obtained by the encoders were sequentially converted into digital data and recorded on a computer for post-match analysis. From the stored data, the distance covered, time spent in the different movement categories and the frequency of occurrence for each activity was determined by Athletic Mode Amisco Pro® (Nice, France) [2], [18], [19]. The following locomotor categories were used: Total distance covered, high intensity running (>14 km⋅h^−1^) and sprinting (>24 km⋅h^−1^), which are similar to movement categories described by others [2], [4], [5], [20]. The multiple-camera match analysis approach has been shown to be a precise method of assessing game activities in football [18] and is programmed to detect work rate (distance covered within different speed thresholds) changes throughout a game [19]. Correlation analyses were performed on match activities and the physiological response during the games. In addition to the movement pattern assessment, technical parameters were determined using the multiple-camera match analysis approach as previously described [5], [20].

### Physiological Measurements

Heart rate was recorded throughout the games (Polar Team system 2, Polar Electro, Kempele, Finland). The maximal heart rate of the participants recorded during the Yo-Yo IR1 (see [17]) was 189±2 bts⋅min^−1^.

Quadriceps muscle temperature (T_m_) was determined in the medial part of vastus lateralis at a depth of 3 cm adjusting for the thickness of the skin by a needle thermistor (MKA08050-A, Ellab A/S, Rødovre, Denmark), with a precision of 0.1°C as previously reported by Mohr et al. [6]. All thermistors were calibrated against a mercury thermometer. Prior to insertion of the sterile needle thermistors, the skin was disinfected by ethanol (70%). T_m_ was determined in 12 players representing all outfield playing positions immediately after each of the two halves. Core temperature (T_c_) was monitored via a wireless ingestible thermometer pill (VitalSense, precision 0.01°C, Mini Mitter, Respironics, Herrsching, Germany) swallowed early in the morning before breakfast, to maximize the transit time before the experimental games. However, due to technical failures, this was substituted by rectal measures using clinical electronic rectal thermometers (Philips, HF, 365, China – precision 0.01°C) at a depth of ∼2 cm following the first and second half of the CON game in 10 of the 17 participants. Ingestible pills have been proven to be a viable alternative to rectal temperature [21] and validated as displaying similar values and circadian variation as rectal temperature [22].

Blood was drawn according to standard procedures from an antecubital vein into heparinized Plasma-Vacutainers (Becton Dickinson, USA). Blood was taken directly before and immediately after the experimental matches. The samples were then stored on ice and transported within 15 minutes to the laboratory. After centrifugation the plasma was analyzed directly for lactate, glucose and sodium on a Cobas b221 Blood Gas Analyzer (Roche, Mannheim, Germany). The analytical variation, expressed as Percentage of Coefficient of Variation (CV%) was 1.5% for lactate, 3.5% for glucose and 0.4% for sodium.

Fluid loss in each player was also determined after voiding nude body mass was measured before the warm-up and after the game (TANITA HD-316, Tokyo, Japan) and the individual fluid intake during the game was accounted for. The players consumed a standardized diet prepared by a nutritionist during the days preceding both games, but only water was provided during the game. The sweat rate was calculated based on the total fluid loss, correcting for urinal loss and fluid ingested.

### Repeated Sprint Testing

Within 2 to 5 minutes after the end of the game, players carried out a repeated sprint test composed of 3×30 m straight-line sprints interspersed with 25 seconds of active recovery (easy jog back to the start line) [2], [6]. The test was performed indoors in a thermo-neutral environment (∼20°C) on an indoor track. Sprinting time was measured by photocell gates placed 1.0 m above ground level (Polifemo Radio Light, Micro Gate, Bolzano, Italy). Each sprint was initiated from a standing position with the arms raised to chest height, 20 cm behind the photocell gate which started a digital timer as described by Mohr et al [2], [6]. The time of each sprint was recorded and reported. The players were familiarized with the test prior to commencement of the study.

### Statistics

Values are presented as mean ± SEM. Differences in activity parameters, heart rate response, blood lactate, fluid loss and sprint performance between CON and HOT games were determined using the paired Student’s t-test including Bonferroni correction. Differences between the two halves, as well as 5 and 15-minute periods within the match in movement activities and body temperatures, were determined using an analysis of variance test with two-way repeated measurements (time x condition). In the case of a significant difference between time periods, a Tukey post hoc test was used to identify the points of difference. The relationship between selected variables was evaluated using Pearson’s product-moment correlation coefficient. Significance level was set as P<0.05.

## Results

### Body Temperature

**Figure 1:**
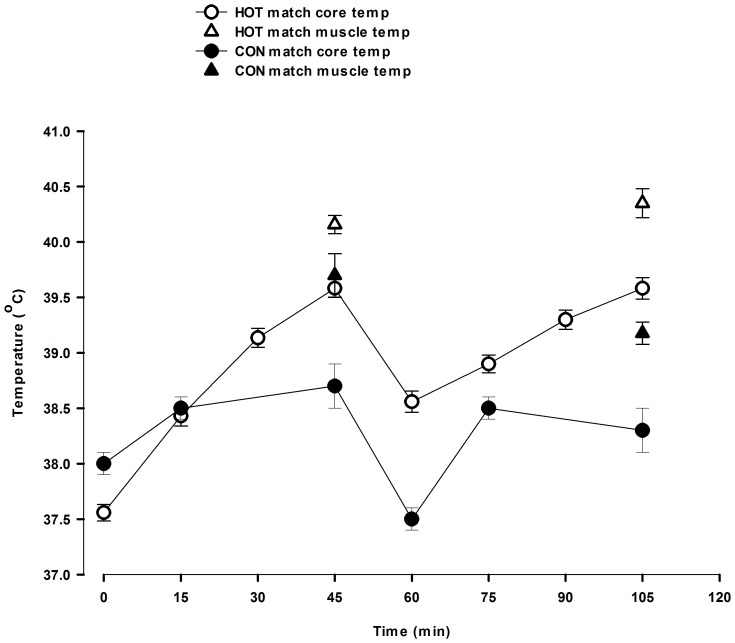
Muscle temperature (Tm; triangles; N = 12) and core temperature (Tc; circles; N = 17) after the first and second half in HOT (open symbols) and CON (closed symbols).

Tc was higher in HOT than CON (Figure 1, P<0.05), both after the 1^st^ half (39.6±0.1 vs. 38.7±0.2°C) and 2^nd^ half (39.6±0.1 vs. 38.3±0.1°C) of the game. Peak Tc was 39.7±0.1°C in HOT and higher (P<0.05) than in CON (38.8±0.2°C). Tc was higher (P<0.05) after the 1^st^ half than after the 2^nd^ half in CON, while Tc tended (P = 0.058) to be higher after the 2^nd^ than after the 1^st^ half in HOT (Figure 1). Tm was not significantly different between trials after the 1^st^ half (40.2±0.1°C in HOT and 39.7±0.2 in CON°C), but was higher in HOT than CON after the 2^nd^ half (40.4±0.0°C vs. 39.2±0.1°C, P<0.05) (Figure 1). Peak Tm was higher in HOT than CON (40.4±0.1°C vs. 39.8±0.2°C, P<0.05). Tm was higher (P<0.05) after the 1^st^ half than after the 2^nd^ half in CON, while there was no difference between halves in HOT (Figure 1).

### Performance Profile

**Figure 2:**
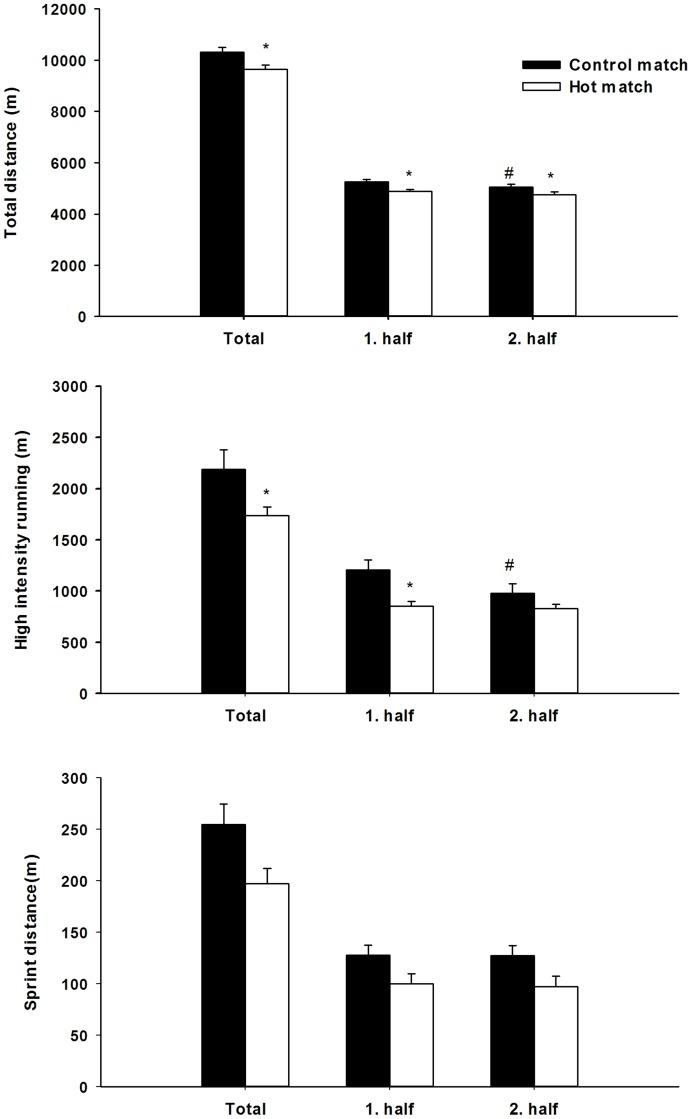
Total distance covered (A), high intensity running (i.e. >14 km⋅h^−1^) (B) and sprinting (i.e. >24 km⋅h^−1^) (C) in total and in each half of CON (black bars) and HOT (white bars). N = 17. *Significant different between conditions; #significant different from the first half (P<0.05).

Total distance covered was 7% shorter (P<0.05) in HOT compared to CON (Figure 2A). A greater distance was covered (P<0.05) in the 1^st^ than in the 2^nd^ half in both HOT (2.7%) and CON (4.2%). In both halves less distance was covered in HOT than in CON (Figure 2A). High intensity running distance was 26% shorter (P<0.05) in HOT than in CON (Figure 2B). There was no difference between halves in HOT while in CON the players ran less (23.5%; P<0.05) at high intensities in the 2^nd^ than in the 1^st^ half (Figure 2B). Players performed 41% less (P<0.05) high intensity running in the first half in HOT compared to CON, with no differences between game trials in the second half (HOT: 883±45 m and CON: 978±97) (Figure 2B). No difference was observed between HOT and CON in total sprint distance (Figure 2C). Moreover, no difference in sprint distance between game trials in either the 1^st^ or the 2^nd^ half of the game was evident (Figure 2C).

In CON, more (P<0.05) high intensity running was performed during the first four 15-minute intervals (range: 361–422 m) than in the final 15-minutes (300±33 m), while no difference was evident between 15-minute periods in HOT (range: 249–350 m). Thus, the P-FI_1_ was 28.0±5.6% in CON, which was higher (P<0.05) than in HOT (−7.2±12.3%). The PFI_2_ was also higher (P<0.05) in CON than in HOT (36.9±8.2% vs. −0.9±9.7%). Sprint distance was not significantly different between the 15-minute periods of the game in either HOT (range: 29–47 m) or CON (range 30–45 m). No significant difference was evident between the two games in sprinting distance in 15-minute intervals of the game.

**Figure 3:**
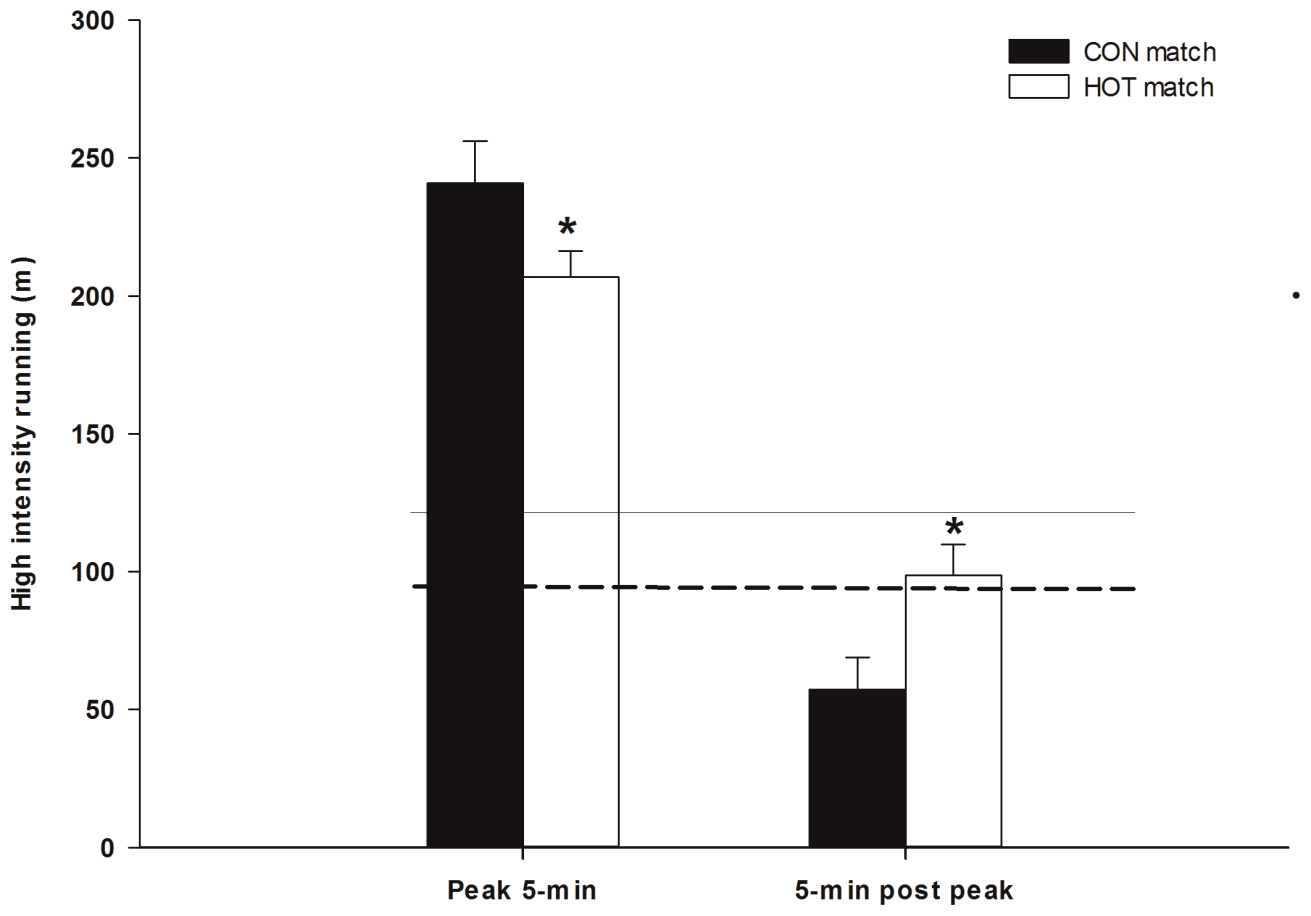
High intensity running during the most intense 5-min period of the game and the 5-min period immediately following the most intense 5-min periods in CON (black bars) and HOT (white bars). Average for all other 5-min periods are shown as the solid and dashed horizontal line for CON and HOT, respectively. N = 17. *Significant different between conditions (P<0.05).

Less (16%; P<0.05) high intensity running was performed during the most intense 5-minute periods in HOT compared with CON (207±9 vs. 241±15 m). However, in the 5-minute period immediately after the most intense 5-minute interval, 74% more (P<0.05) high intensity distance was covered in HOT compared to CON (99±11 vs. 57±12 m, respectively) (Figure 3). In CON, the amount of high intensity distance in the 5-minute period following the most intense periods was 114% lower (P<0.05) than the average distance covered at high intensities in all other 5-minute intervals, with no significant difference in HOT (Figure 3). The T-FI was lower (P<0.05) in HOT (50.6±5.8%) than in CON (78.1±3.7%).

Peak sprinting speed reached in the game was 4% faster (P<0.05) in HOT (33.3±0.4 km⋅h^−1^) than in CON (32.1±0.4 km⋅h^−1^). There was no difference in sprint frequency (11±1 and 13±1) and length (19.6±0.7 and 19.9±0.8 m) between HOT and CON respectively. Peak speed reached in a sprint was higher (P<0.05) in HOT than CON in the first, third and fourth 15-minute interval (Table 1). Average sprinting speed was also higher (P<0.05) in HOT in comparison to CON in the first 15-minutes of each half (Table 1). No significant difference in average length of sprint in 15-minute intervals between HOT and CON (Table 1) was evident.

**Table 1:** Sprint characteristics in 15-min periods in HOT and CON.

	0–15		15–30		30–45		45–60		60–75		75–90	
**Peak Speed**												
** CON**	28.7	0.3	29.3	0.3	28.2	0.2	27.7	0.3	28.6	0.5	28.6	0.4
** HOT**	30.2*	0.3	29.1	0.4	30.1*	0.6	29.8*	0.5	29.6	0.5	28.9	0.6
**Average Speed**												
** CON**	25.2	0.1	24.9	0.2	25.3	0.3	24.9	0.2	25.1	0.2	25.3	0.1
** HOT**	26.1*	0.2	25.9	0.2	25.7	0.3	26.5*	0.2	26.0	0.3	25.6	0.4
**Length**												
** CON**	18.6	1.5	23.9	1.3	20.7	1.7	20.4	1.9	20.0	1.3	21.8	2.0
** HOT**	20.0	1.2	19.2	1.3	19.7	1.7	20.0	1.9	20.6	1.4	19.0	1.6

Average peak speed (km·h^−1^), average speed (km·h^−1^) and average length (m) of the sprints in the game in 15-min intervals (N = 17).

* Significant different between conditions (P<0.05).

### Technical Parameters

**Figure 4:**
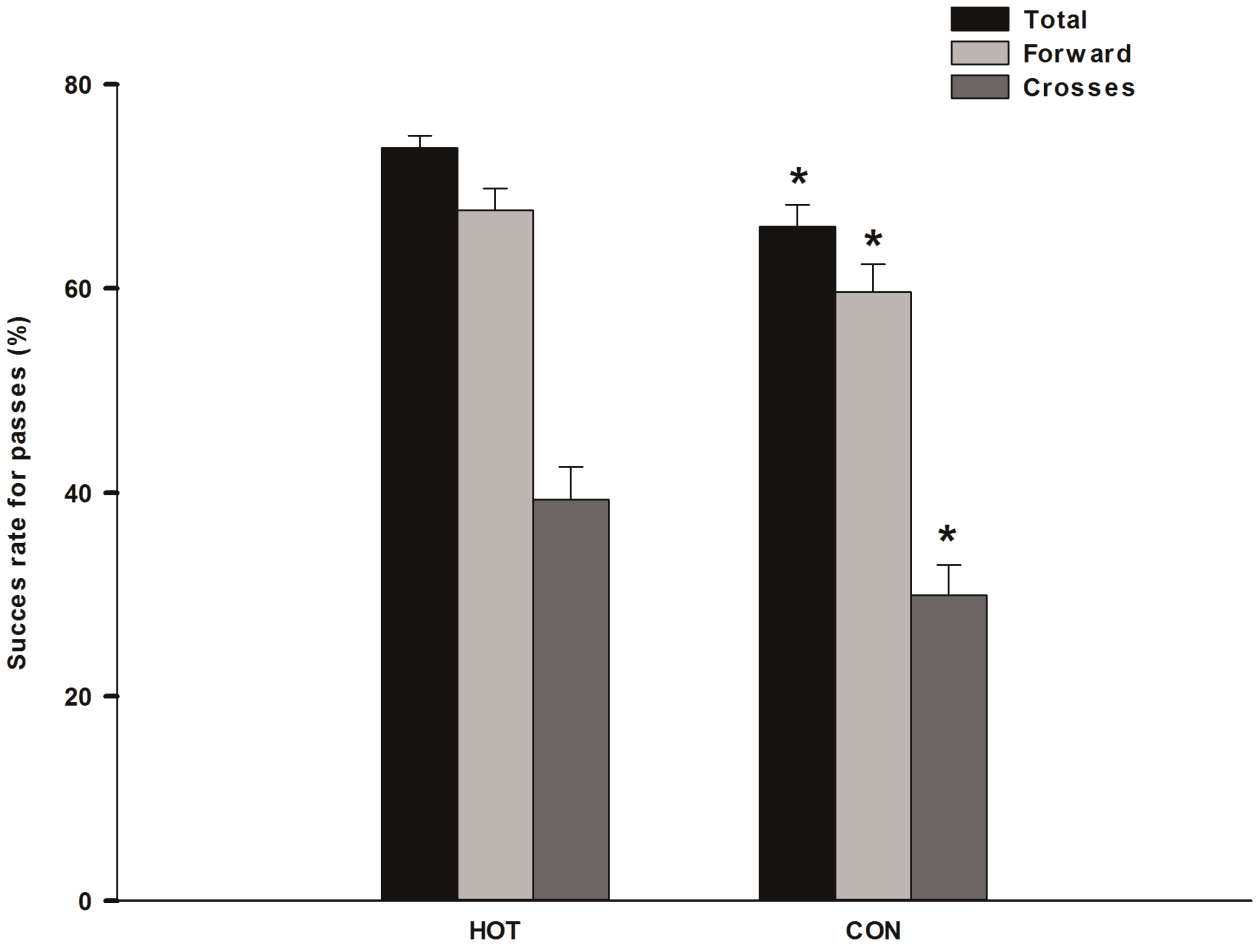
Success rate of all passes (black bars), forward passes (light grey bars) and crosses (dark grey bars). N = 17. *Significant different between conditions (P<0.05).

In HOT there was a higher success rate for all passes (73.8±1.2 vs. 66.1±2.1%) and forward passes (67.7±2.1 vs. 59.7±2.7%, respectively) (Figure 4) compared to CON (both P<0.05). Additionally the success rate for crosses in HOT was higher (P<0.05) than in CON (39.3±3.2 vs. 29.9±3.0%, respectively) (Figure 4). There was no significant difference in the total number of passes, forward passes, crosses or average length of passes and crosses performed in HOT and CON. The higher success rate of passes and crosses implies increased ball possession in HOT compared to CON (26.5 vs. 21.2 min, respectively). Conversely, both gain and loss of ball possession were higher (P<0.05) in CON (14.0±1.3 and 21.1±1.5, respectively) in comparison to HOT (6.4±0.8 and 9.8±1.1, respectively). Additionally, the average number of challenges was higher (P<0.05) in CON than in HOT (5.8±0.3 vs. 4.2±0.3). The average sum of ground and air duels was also higher (P<0.05) in CON (2.2±0.2 and 1.8±0.1, respectively) than HOT (1.4±0.2 and 0.8±0.1, respectively).

### Heart Rate Response

Average heart rate during the HOT and CON games was 158±2 and 160±2 bts⋅min^−1^, respectively, corresponding to 83.7±0.8 and 84.6±0.8% of the maximal heart rate. The peak heart rate reached during the games was 185±3 and 183±2 bts⋅min^−1^ in HOT and CON, respectively, which is 97.7±0.7 and 97.0±0.8% of the maximal heart rate. There was no significant difference in average or peak heart rate between the HOT and CON trials.

### Plasma Lactate, Glucose and Sodium

The plasma lactate concentration was 1.7±0.1 and 1.6±0.1 mmol⋅l^−1^ prior to HOT and CON and increased (P<0.05) to 4.9±0.5 and 3.3±0.4 mmol⋅l^−1^ immediately after the games, respectively, with no significant difference between trials. Plasma glucose was 5.0±0.2 and 5.3±0.1 mmol⋅l^−1^ prior to HOT and CON, respectively and increased (P<0.05) to 6.8±0.2 and 6.4±0.2 mmol⋅l^−1^ after the games with no significant difference between HOT and CON. The plasma Na^+^ concentration increased (P<0.05) from 138±0 to 142±1 mmol⋅l^−1^ during the HOT game trial and from 138±3 to 140±1 mmol⋅l^−1^ in CON, but was not different across conditions.

### Fluid Loss and Intake

Total sweat volume was 4.14±0.16 L in HOT, which was higher (P<0.05) than CON (2.59±0.14 L) and corresponded to average sweat rates of 2.48±0.09 and 1.55±0.09 L⋅h^−1^, respectively. The water intake was greater (P<0.05) during HOT than CON (2.6±0.2 L vs. 1.1±0.1 L, respectively) making the game-induced decrease in body mass similar in HOT and CON (1.9±0.3 and 1.8±0.1%, respectively). The percentage decrease in total game distance from CON to HOT was not correlated (r = 0.34, P = 0.16) to the game-induced loss in body mass.

### Repeated Sprint Performance

**Figure 5:**
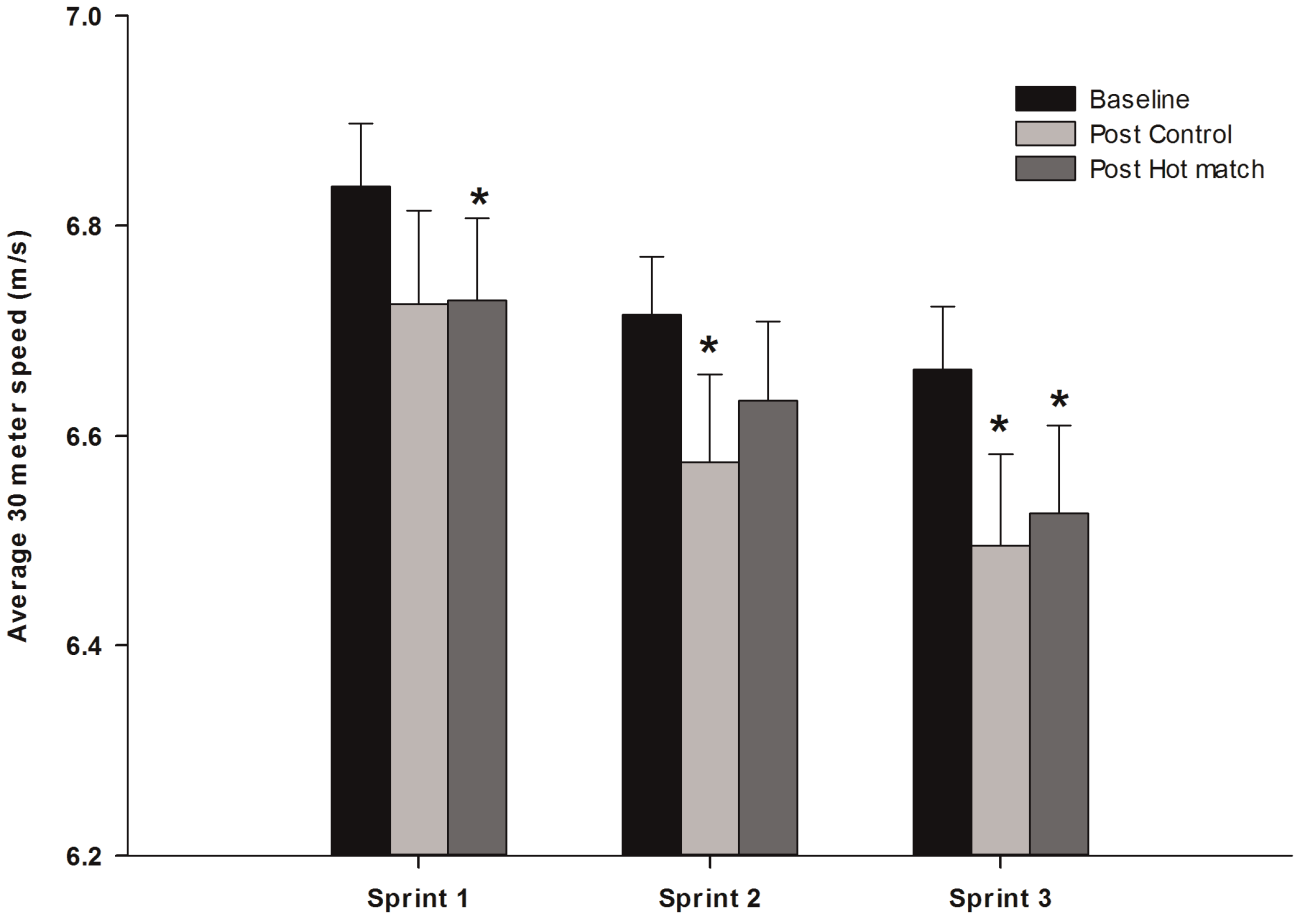
Average 30-m sprinting speed in baseline (black bars), after CON (light grey bars) and after HOT (dark grey bars). N = 17. *Significant different from baseline (P<0.05).

Compared to baseline, sprint performance after both games was reduced (P<0.05), but there were no significant differences in post-game repeated sprint performance between HOT and CON (Figure 5). Total sprint test time was 13.36±0.11 seconds in a rested state before the experimental games and decreased (P<0.05) by 1.7 and 2.1% respectively after HOT and CON. The 1^st^ and 3^rd^ sprints were slower (P<0.05) after HOT compared to baseline, while the 2^nd^ and 3^rd^ sprints were slower (P<0.05) in CON (Figure 5).

### Correlations

**Figure 6:**
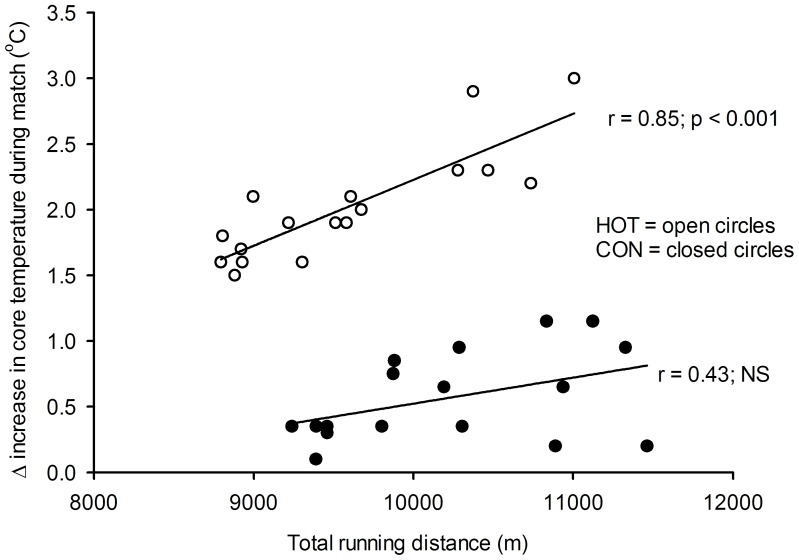
Individual relationship between total distance covered during HOT and CON and delta increase in core temperature (°C) where open circles are from the HOT game and the closed circles are from CON.

Delta increase in core temperature in HOT was correlated (r = 0.85; P<0.05) to the total distance covered (Figure 6), but not in CON (r = 0.43; NS). Absolute core temperature at the end of HOT was also correlated (r = 0.53; P<0.05) to total distance covered and distance covered in the last 15 minutes of the HOT game (r = 0.48; P<0.05). The relative distance-deficit between CON and HOT was not correlated to peak core temperature, while the delta increase in core temperature from CON to HOT did not correlate to percentage reduction in total distance covered from CON to HOT. Also, plasma lactate concentration at the end of HOT was not correlated to percentage reduction in total distance covered from CON to HOT. Core temperature was not correlated to neither T-FI nor P-FI_1_ and PFI_2_ in the HOT game. Total distance covered and high intensity running during CON was correlated (r = 0.79 and 0.76, respectively; P<0.05) with Yo-Yo IR1 performance, whereas in HOT, high intensity running correlated to Yo-Yo IR1 performance (r = 0.65, P<0.05), but not total distance covered (r = 0.37, NS). The P-FI_1_ and P-FI_2_ in CON correlated inversely with Yo-Yo IR1 performance (r = −0.69 and r = −0.50, respectively; P<0.05), but not in HOT. Total sprint time in the baseline repeated sprint test correlated inversely (r = −0.53; P<0.05) with high intensity running in CON, but not in HOT.

## Discussion

Our data showed that well-trained football players have a moderate reduction in total distance (7%) and a marked decline (26%) in the amount of high intensity running completed when a game is played in hot ambient conditions compared to a temperate environment. In contrast, peak sprint velocity was higher in the heat and the technical quality, as indicated by percentage of successful passes and ball possession, was enhanced rather than impaired. The reductions in total and high intensity running were not correlated to absolute or delta changes in core or muscle temperature. Neither changes in plasma lactate, heart rate nor the net-loss of body mass could predict the hyperthermia-induced decline in work performance. In HOT the players who achieved the highest core temperatures were those with the highest work capacity, whereas there was no relationship between core temperature changes and work performance in the control game.

The altered pacing strategy in the HOT game is in accordance with responses observed when games are completed under moderate heat stress [1]. In the present study the movement patterns throughout the game were studied in detail and showed several discrepancies in different match performance variables between the two game trials. For example, in the CON game there was a greater distance deficit in high intensity running in the second half compared to the first half which did not occur in the HOT game. The observation of a higher exercise intensity in the first compared to the second half in games played in thermo-neutral conditions agrees with numerous studies [4], [5], [18], [23]. A possible explanation for the different responses in CON and HOT is that the high work rate in the first half of the CON game will deteriorate physical performance in the second half, which is supported by Rampinini et al. [24]. On the other hand, the pacing strategy was markedly different in HOT with a relatively low work rate during the first half (41% less high intensity running than in CON) potentially leading to less accumulative fatigue. Consequently, players were able to achieve similar absolute exercise intensity as the second half in the CON game, despite a 23°C warmer ambient temperature and markedly higher body temperatures. Another suggestion is that the players change their behavior tactically in the heat analogously to the changes in the duration of duels and rest between duels during tennis in environmentally challenging situations [25] and by anticipatory altered pacing during time-trial cycling in heat [12], [26].

In addition to a more constant but lower running intensity in HOT than CON, the technical data substantiated large differences between the two games. Indeed, time in ball possession was ∼25% higher and the success rate of passes, forward passes and crosses was significantly greater in HOT compared to CON. In contrast, gain and loss of ball possession as well as the number of duels was higher in the CON trial, indicating a more transition-based type of play in CON compared to the more possession-orientated play in HOT. Others have shown that technical variables are affected by playing standard [27], surface [23], field dimensions [28], playing formation [5] and physiological fatigue [20]. In the present study the same players, similar turf and field dimension as well as the same type of playing formation were applied in both game-trials. This suggests that the higher running intensity in CON is likely to have provoked a higher number of technical mistakes. In part this might be consequent to an increase in pressure from the opposition when in ball possession and due to a greater degree of transient muscle fatigue throughout the CON game. Hyperthermia may affect cognitive performance during complex tasks [29], however it appears that any negative effect of heat stress on the players technical skills was compensated for by the lower amount of high intensity running and hence less pressure on the player in ball possession.

In normal temperate conditions fatigue has been shown to develop in the final 15-minute interval of competitive high level games [4], [16] as well as in a simulated football protocol [30]. The P-FI_1_ and P-FI_2_ represent the ability to exercise at a high intensity during the final 15-minutes of a game, compared to earlier in the game [2]. These indicators of accumulative game-induced fatigue or altered pacing were noticeable higher in CON than HOT. Thus the exercise intensity during the CON game resulted in a much more pronounced decline in running intensity in the final and critical stage of the CON game compared to HOT. However, despite the markedly different pacing strategy in HOT than in CON, repeated sprint test performance was equally compromised after the games irrespective of the ambient temperature. This indicates that the overall fatigue response after the two game trials was the same. In addition, during the most intense 5-minute period of the game, where temporary fatigue occurs that mandates a short recovery [4], [5], [23], [30], [31], 16% more high intensity running was performed in CON than in HOT. This created a greater decline in high intensity running in the following 5-minute interval and produced a concomitant higher T-FI in CON compared to HOT (Figure 3). Therefore, the higher exercise intensity in CON also appears to increase the degree of the more transient type of performance decrement [16] in CON compared to the HOT game. This notion is further supported by the fact that the total sprint distance was the same in CON and HOT, but the peak and average sprint speed was higher in HOT than in CON. Hence the pacing patterns appear to be greatly affected by hot ambient temperatures, resulting in markedly lower but more constant exercise intensity with improved sprinting quality in comparison to a temperate environment.

In further support of hyperthermia-induced effects on the pacing strategy in HOT is the fact that Yo-Yo IR1 performance was correlated to the total distance covered and both P-FIs in CON, but not in HOT. Thus the pacing in football can partly be predicted from training status at normal temperatures but less in severe heat where other physiological (e.g. thermal) and behavioral (i.e. pacing) mechanisms [9], [11], [12], [14], [25], [26], [32] might dictate the limitations for physical performance.

In the present study, core temperature was more than 1°C higher in HOT than in CON. The core temperature levels were above 39°C during most of the HOT game and peak levels exceeded 39.5°C which is similar to temperatures reported previously in a football game in hyperthermic conditions [3]. The increase in core temperature was correlated to the total game distance in the HOT game, but the distance deficit in high intensity running from CON to HOT was not directly correlated to the player’s peak core temperature or the delta increase in core temperature from CON to HOT. Therefore, physical activity during a hot game is not simply a function of absolute core temperature, but may potentially be dictated by individual differences in the response to and the perception of heat, leading to different pacing strategies.

While body core hyperthermia may hamper prolonged exercise performance or central activation during repeated sprints [7]–[9], [13], [14], a potential benefit of the elevated muscle temperature may be gained. Specifically, increased muscle temperature has been shown to enhance sprint performance during a football game [6]. Our data showed that hot ambient conditions have a variable impact on sprint performance since the total sprint distance was the same in HOT and CON, whereas the peak and average speed was higher in HOT than CON. Several studies have reported improved muscle contractile properties [33] leading to higher power production and, in turn, better performance [34] in hot compared to neutral ambient conditions. This seems to be specifically the case in the morning, before the diurnal increase in body temperatures (due to circadian rhythms) acts as a warm-up in the late afternoon [35]. In the current study, the kickoff of both games was at 1100 hours confirming the potential positive effect of hot ambient temperature on sprint capacity at this time of day. In addition the average speed in the sprints was higher in HOT than in CON during the first 15 minutes of each half where the core temperature was relatively low, indicating that elevated muscle temperature improves sprint ability when core temperature is maintained below ∼39°C.

It is also likely that the lower average intensity in the most intense periods of the game in HOT stressed peripheral fatiguing mechanisms such as muscle acidosis and, to a lesser extent, extracellular potassium accumulations [7], [8]. Therefore, the decreased work rate in the hot environment probably lowered the level of peripheral fatigue which in turn might be beneficial for sprint performance during the game. However in the present study, blood lactate concentrations after the HOT game were not lower than after CON, suggesting that glycolytic activity in the heat was at least as high as the control game despite the reduced amount of high intensity running. Additionally, the muscle glycogen levels measured in muscle biopsies obtained immediately after both games were similar in HOT and CON i.e. higher than 250 mmol⋅kg^−1^ d.w. (data not shown). Therefore it is unlikely that the degree of depleted muscle glycogen stores contributed to the differences in match performance between HOT and CON.

High fluid-loss may affect both the capacity to perform prolonged submaximal and intense exercise [36]. However in the present study, the net-fluid loss was only moderate (<2% of body mass) and was similar in HOT and CON due to a markedly higher fluid intake in the hot game. Interestingly, when *ad libitum* water was provided to the players, they voluntarily drank a sufficient amount to maintain their body weight. Additionally, the percentage loss of body mass in HOT did not correlate to the distance-deficit from CON to HOT. Taken together, these observations suggest that the lower exercise intensity was not mediated by dehydration, which also is supported by others [37]. The sweat rate was very high in HOT and is in line with recent reports from high level competitive football games played at high environmental temperatures [38].

In conclusion, the present study shows, for the first time with detailed analysis of the players activity patterns, how competitive football in a hot environment affects the various component of physical performance. Heat stress lowers the average work rate in the game, as well as high intensity efforts, but reduces intensity fluctuations throughout the game and increases peak sprinting speed and certain technical parameters of the game. The core and muscle temperature changes were not strong predictors of the performance changes in hot ambient condition.
